# Behavioral, climatic, and environmental risk factors for Zika and Chikungunya virus infections in Rio de Janeiro, Brazil, 2015-16

**DOI:** 10.1371/journal.pone.0188002

**Published:** 2017-11-16

**Authors:** Trevon L. Fuller, Guilherme Calvet, Camila Genaro Estevam, Jussara Rafael Angelo, Gbenga J. Abiodun, Umme-Aiman Halai, Bianca De Santis, Patricia Carvalho Sequeira, Eliane Machado Araujo, Simone Alves Sampaio, Marco Cesar Lima de Mendonça, Allison Fabri, Rita Maria Ribeiro, Ryan Harrigan, Thomas B. Smith, Claudia Raja Gabaglia, Patrícia Brasil, Ana Maria Bispo de Filippis, Karin Nielsen-Saines

**Affiliations:** 1 Institute of the Environment and Sustainability, University of California Los Angeles, Los Angeles, California, United States of America; 2 Instituto Nacional de Infectologia Evandro Chagas, Fundação Oswaldo Cruz, Rio de Janeiro, Brazil; 3 Universidade Estadual de São Paulo, Rio Claro, São Paulo, Brazil; 4 Escola Nacional de Saúde Pública, Fundação Oswaldo Cruz, Rio de Janeiro, Brazil; 5 Foundation for Professional Development, Pretoria, Gauteng, South Africa; 6 David Geffen UCLA School of Medicine, Los Angeles, California, United States of America; 7 Laboratorio de Referência de Flavivirus, Instituto Oswaldo Cruz, Fundação Oswaldo Cruz, Rio de Janeiro, Brazil; 8 Department of Ecology and Evolutionary Biology, University of California Los Angeles, Los Angeles, California, United States of America; 9 Biomedical Research Institute of Southern California, Oceanside, California, United States of America; Instituut voor Tropische Geneeskunde, BELGIUM

## Abstract

The burden of arboviruses in the Americas is high and may result in long-term sequelae with infants disabled by Zika virus infection (ZIKV) and arthritis caused by infection with Chikungunya virus (CHIKV). We aimed to identify environmental drivers of arbovirus epidemics to predict where the next epidemics will occur and prioritize municipalities for vector control and eventual vaccination. We screened sera and urine samples (*n* = 10,459) from residents of 48 municipalities in the state of Rio de Janeiro for CHIKV, dengue virus (DENV), and ZIKV by molecular PCR diagnostics. Further, we assessed the spatial pattern of arbovirus incidence at the municipal and neighborhood scales and the timing of epidemics and major rainfall events. Lab-confirmed cases included 1,717 infections with ZIKV (43.8%) and 2,170 with CHIKV (55.4%) and only 29 (<1%) with DENV. ZIKV incidence was greater in neighborhoods with little access to municipal water infrastructure (*r* = -0.47, *p* = 1.2x10^-8^). CHIKV incidence was weakly correlated with urbanization (*r* = 0.2, *p* = 0.02). Rains began in October 2015 and were followed one month later by the largest wave of ZIKV epidemic. ZIKV cases markedly declined in February 2016, which coincided with the start of a CHIKV outbreak. Rainfall predicted ZIKV and CHIKV with a lead time of 3 weeks each time. The association between rainfall and epidemics reflects vector ecology as the larval stages of *Aedes aegypti* require pools of water to develop. The temporal dynamics of ZIKV and CHIKV may be explained by the shorter incubation period of the viruses in the mosquito vector; 2 days for CHIKV versus 10 days for ZIKV.

## Introduction

The burden of arboviral disease in the Americas is high and increasing. It includes infants permanently disabled by infection with the Zika virus (ZIKV) in Brazil [1–3] and persistent, incapacitating arthritis caused by infection with Chikungunya virus (CHIKV) [4]. The past decade has seen a substantial increase in the burden of arboviruses driven by factors such as the proliferation of mosquito breeding sites in cities and range expansions of ZIKV and CHIKV from Africa and Asia to Oceania and the Americas [5–9]. It is predicted that with climate the ranges of *Aedes* mosquitoes that are vectors of CHIKV, DENV, and ZIKV will expand in South America [10].

Although a variety of studies have investigated the introduction of a single arbovirus into a naïve population, an integrative analysis of more than one arbovirus has the potential to yield insights about interactions among the viruses. A salient example is the state of Rio de Janeiro, Brazil, which has experienced recent epidemics of four arboviruses transmitted by the mosquito *Aedes aegypti*: CHIKV, dengue (DENV), Yellow Fever virus, and ZIKV. ZIKV is of particular concern because in pregnant women infection can result in fetal abnormalities including microcephaly [11, 12]. In non-pregnant adults approximately 1% of ZIKV infections result in Guillain-Barré Syndrome (GBS), an inflammatory disorder that causes acute flaccid paralysis [13, 14]. Like ZIKV, CHIKV is a novel arbovirus in Rio de Janeiro with the first locally transmitted cases reported in 2015 [15].

As a variety of ecological and economic factors could contribute to arbovirus epidemics in Rio de Janeiro including sanitation infrastructure and pools of standing water that become mosquito breeding sites, an in-depth study is needed to assess the importance of these factors. To understand arboviruses in an integrative fashion, the objectives of the present study were to 1) identify drivers of CHIKV and ZIKV epidemics. Although we included DENV in the analysis because it is endemic in Rio de Janeiro [16], the focus of our study was CHIKV and ZIKV as they are novel arboviruses in the region and caused epidemics during the study period; 2) predict where the next epidemics may occur; and 3) prioritize areas for vector control and eventual vaccination. We screened suspected cases by polymerase chain reaction (PCR), and clinical criteria and analyzed the timing, geographic locations, and socio-economic and infrastructure-related characteristics of confirmed cases.

## Materials and methods

We used an ecological study design to collect serum and urine samples from symptomatic patients suspected to have CHIKV, DENV, or ZIKV in the state of Rio de Janeiro (*n* = 10,459). Samples were collected at outpatient clinics and hospitals in 48 municipalities across the state of Rio de Janeiro from January 2015 to October 2016 (S1 Table). None of the patients had a history of travel outside the state. Patients were offered the opportunity to participate in the study if they were suspected to have an arbovirus based on clinical signs and symptoms such as acute febrile illness with mosquito exposure. After providing written informed consent, participants filled out an information sheet with their age, sex, and the municipality and neighborhood where they lived. Human subject research was approved by the Evandro Chagas National Institute of Infectious Diseases, Oswaldo Cruz Foundation (Ethics Approval CAAE0026.0.009.000–07). Sera and urine were tested for ZIKV by real-time RT–PCR with the QuantiTect Probe kit [17, 18] and for DENV using the CDC RT-PCR Assay [19]. Sera and urine were tested for CHIKV by qRT-PCR [20] or by enzyme-linked immunosorbent assay (ELISA) to detect IgM antibodies using the Euroimmun kit [21, 22]. A confirmed case was anyone positive for ZIKV by qRT-PCR, positive for DENV by qRT-PCR, or positive for CHIKV either by qRT-PCR or by ELISA.

If both sera and urine samples from the same individual were positive by lab assays, to avoid duplicating cases, only one positive sample was retained for climatic and spatial modeling. Furthermore, if a patient was positive for CHIKV by RT-PCR and IgM, we only included the date of the RT-PCR positive sample in the database, because the date of the PCR positive is closer to the acute phase of the patient’s illness. Finally, if a patient tested negative for CHIKV by PCR but was positive by IgM, the IgM sample was included in the database of positive samples.

Screening was conducted at the Brazilian Ministry of Health Regional Reference Laboratory for CHIKV, DENV, ZIKV, Yellow Fever, and West Nile virus—Instituto Oswaldo Cruz (hereafter LABFLA). No Yellow Fever virus or West Nile virus screening was carried out for this analysis. LABFLA is a centralized reference service that receives samples from all areas of the state of Rio de Janeiro (http://www.fiocruz.br/ioclabs/cgi/cgilua.exe/sys/start.htm?sid=58). Due to its status as a reference center, during the ZIKV and CHIKV epidemic, samples from across the state were submitted to LABFLA. Sample collection was first coordinated at the state of Rio de Janeiro public health lab, city health departments in the municipalities of Campos dos Goytacazes and Niterói and three research institutions: the Evandro Chagas Institute, the Fernandes Figueira Institute, and the National School of Public Health. In addition, we collected samples at the Oswaldo Cruz Foundation clinic in Manguinhos, which is a community with 30,000 inhabitants in the northern district of the city of Rio de Janeiro. Patients were referred to the clinic from other clinics or sought care there as they lived nearby due to clinical suspicion of disease. After collection at the aforementioned institutions (Table 1), the samples were sent to LABFLA for screening.

**Table 1:** Characteristics of the LABFLA data set. ZIKV samples were collected from January 2015 to May 2016, and CHIKV from September 2015 to October 2016.

Institutions that coordinated sample collection
These institutions coordinated the collection of 70% of the samples:
• Central Public Health Laboratory of Rio de Janeiro • Evandro Chagas National Institute of Infectious Diseases, Oswaldo Cruz Foundation • Fernandes Figueira National Institute of Women’s, Children’s, and Adolescent Health, Oswaldo Cruz Foundation • Municipal Health Department of Niterói • Municipal Health Department of Campos dos Goytacazes • National School of Public Health, Oswaldo Cruz Foundation
These institutions coordinated the collection of the remaining 30%:
• Oswaldo Cruz Foundation Clinic in Manguinhos, Rio de Janeiro • Public and private hospitals

In Rio de Janeiro the first locally acquired ZIKV infections were detected in May 2015, however LABFLA conducted a retrospective study of samples that were collected beginning in January 2015, and tested negative for DENV by PCR. The retrospective study detected ZIKV by RT-PCR in February 2015.

Previous studies have compared the diagnosis of ZIKV in Rio de Janeiro based on clinical features versus laboratory tests. The results indicated that the symptoms that best discriminate ZIKV from DENV and CHIKV are pruritus and conjunctival hyperemia [16]. We analyze lab-confirmed cases of CHIKV, DENV, and ZIKV. The use of lab-confirmed cases reduces the geographic scope of the analysis somewhat to the extent that six municipalities in the state of Rio de Janeiro reported clinical cases of CHIKV, DENV, or ZIKV but not lab-confirmed cases. However, the municipalities that reported clinical cases but not lab-confirmed cases are all small towns (average population: 30,000 inhabitants) that do not appear to have experienced large outbreaks of CHIKV, DENV, and ZIKV.

### Climatic, geographic, and infrastructural analysis

As cases of DENV were extremely rare, we investigated how climate and infrastructure affected the timing and geographic pattern of incidence of ZIKV and CHIKV. The scale of the analysis was the state of Rio de Janeiro (area: 44,000 km^2^), which has a warm temperate climate in mountainous areas of the interior and an equatorial climate along the coast [23]. The larval stages of *Aedes aegypti* require shallow pools of water to develop [24]. After a rainstorm, such pools will be more abundant. In light of this, we hypothesized that after a delay of two to three weeks following a major rain event there will be increased mosquito abundance, and heightened vector transmission of ZIKV. To test this hypothesis, we obtained rainfall data from March 2015 to December 2016 from six weather stations operated by the National Institute of Meteorology [25].

Further, we investigated socio-economic risk factors for ZIKV by analyzing data on sanitation in the city of Rio de Janeiro. Previous studies in Rio de Janeiro have shown that lack of access to municipal water prompts households to hoard water in barrels that become infested with *Aedes aegypti* larvae, leading to increased vector abundance and DENV transmission [26]. To assess whether water infrastructure also affects ZIKV risk, we compared the incidence of ZIKV per neighborhood with the percentage of households serviced by the municipal system. Water data were obtained from the GeoOpenData portal of the Rio de Janeiro Mayor’s Office. Water storage is linked to socio-economic status in Brazil insofar as having running water in the household is correlated with income such that low-income households are less likely to be connected to water infrastructure and more likely to store water in improvised containers [27, 28]. We calculated Pearson’s product moment correlation coefficient between CHIKV and ZIKV incidence and variables representing the level of urbanization at the neighborhood scale and the availability of infrastructure such as access to the municipal water system.

We also tested whether there was a correlation between the ZIKV incidence in a municipality and mosquito density. Our analysis used data from the 2016 *Rapid Assessment of Aedes aegypti Infestation* of the State Health Department of Rio de Janeiro, which is a standardized insect survey carried out at the municipal scale. During the survey, trained observers made note of the presence of mosquito larvae and containers where mosquitos can oviposit such as tires filled with water [29].

### Epidemic model

**Fig 1:**
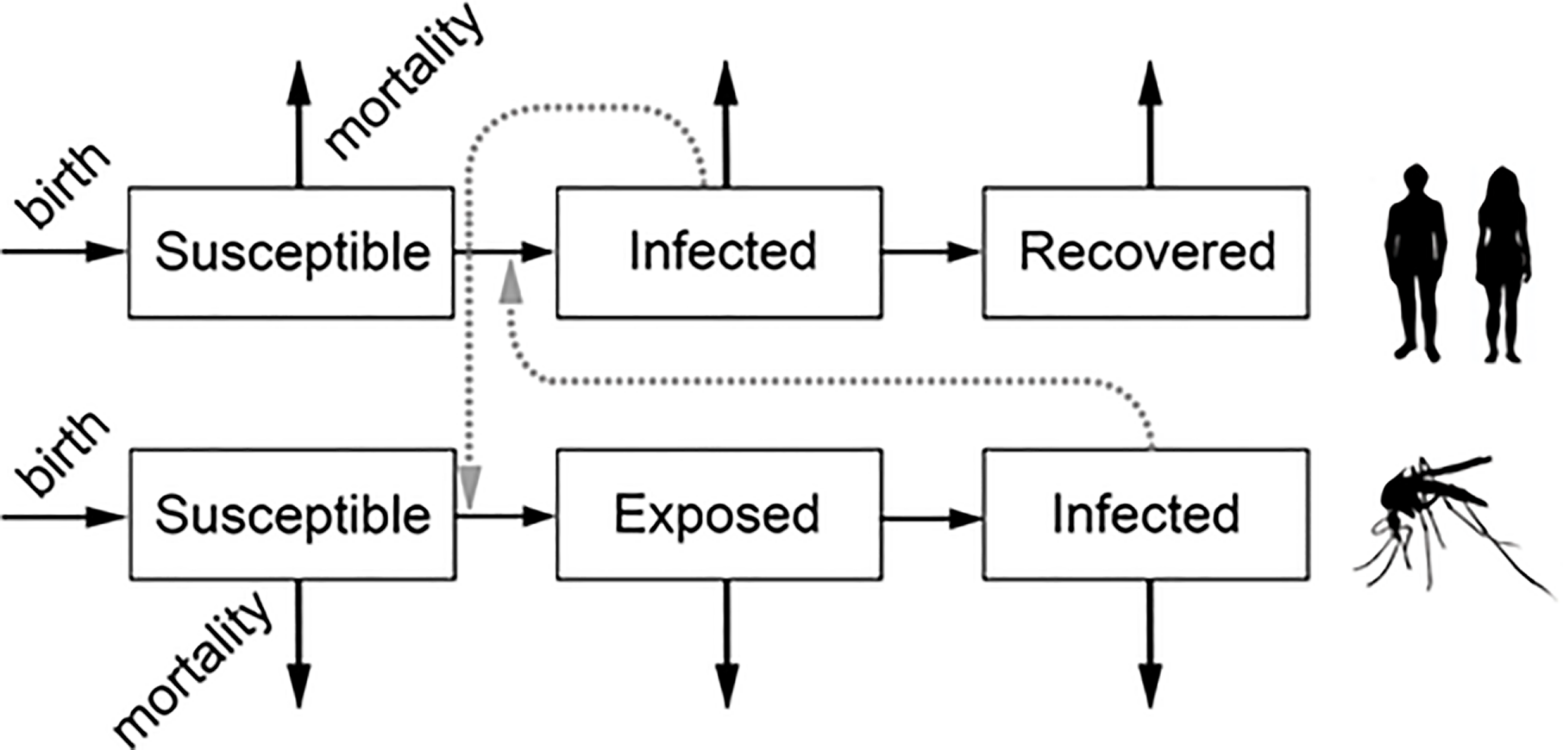
Compartmental model used to simulate CHIKV and ZIKV epidemics.

We developed a simulation model to explore what-if scenarios that might explain the timing of CHIKV and ZIKV epidemics in Rio de Janeiro (S2 Table). The model was based on differential equations like MacDonald-Ross models used to simulate malaria transmission [30–32]. Each arbovirus was modeled independently of the other using the epidemic model (Fig 1). The human population was divided into three compartments: individuals susceptible to infection, those currently infected, and the recovered. The model simplified the transmission cycle by omitting sexual transmission. The mosquito population was divided into susceptible, exposed but not infectious, and infected compartments. We assumed that due to the short lifespan of the vector, it would not recover from the infection. Humans can become infected by being bitten by an infected mosquito. Further, mosquitoes can become infected by biting an infected human. In both the human and mosquito populations, new susceptibles are born into the population and some susceptibles experience mortality due to causes other than the arbovirus.

The simulation model included parameters such as the incubation period of the viruses in the mosquito. As it was not possible to measure these parameters in Rio de Janeiro, we used values from the literature (S3 and S4 Tables). The model was implemented in MatLab using a fourth order Runge-Kutta scheme.

## Results

### Lab-confirmed cases

Of the 10,459 patients screened for CHIKV, DENV, and ZIKV, 6,543 were negative for the three arboviruses. There were 3,887 lab-confirmed cases of CHIKV and ZIKV including 1,717 of ZIKV (44.2%), and 2,170 of CHIKV (55.8%) (Table 1). ZIKV positive samples were detected from February 2015 to May 2016 and CHIKV positive samples from September 2015 to October 2016. Compared to the other two viruses, there were very few lab-confirmed cases of DENV (*n* = 29). As DENV was very rare, we did not analyze the timing or spatial pattern of DENV infections.

### Timing of rainfall, CHIKV, and ZIKV cases

**Fig 2:**
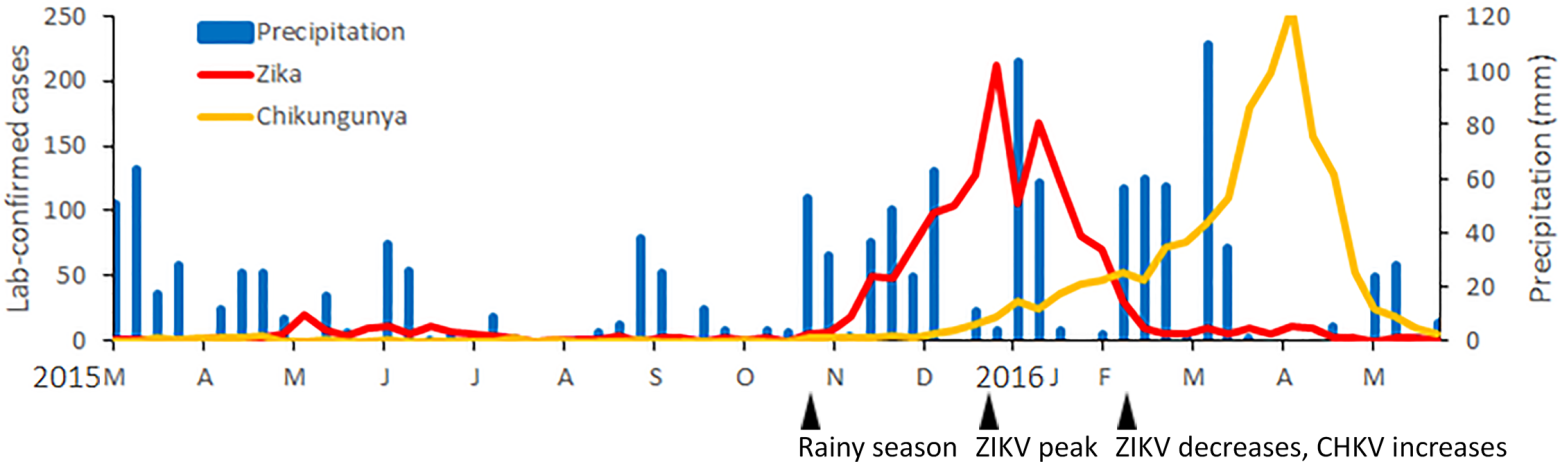
Confirmed cases of ZIKV and CHIKV per week in the state of Rio de Janeiro, March 2015 to May 2016 (LABFLA data set).

From mid-September to early October 2015, a series of large rainstorms occurred, which were followed 3–4 weeks later by the beginning of the largest outbreak of ZIKV, which began in October and continued to December 2015 (Fig 2). The largest wave of the ZIKV epidemic ended in the first quarter of 2016 (Fig 2). The greatest number of cases of ZIKV was reported in January 2016, after which there was a steady decline in cases. Cases of CHIKV were lowest in January and increased steadily every month during the first quarter of 2016. In late February and early March 2016, major rains occurred, after which cases of CHIKV increased, peaking in April. We did not observe a coupling between temperature or relative humidity and ZIKV cases (S3 and S4 Figs).

According to the LABFLA database, the number of cases of CHIKV in February to May 2016 was greater than the number of cases of ZIKV.

### Socio-economic and geographic characteristics of lab-confirmed cases of ZIKV and CHIKV

**Fig 3:**
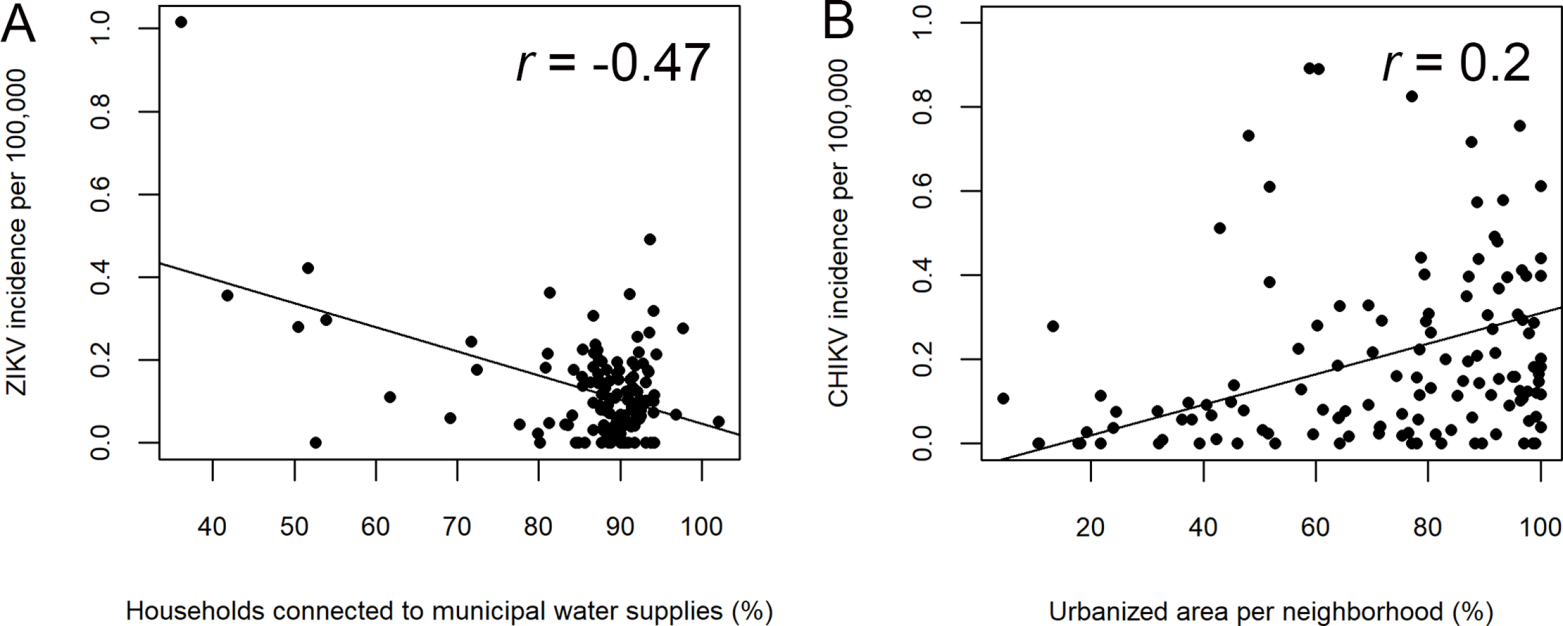
Effect of infrastructure on CHIKV and ZIKV incidence in the city of Rio de Janeiro. Each point represents one neighborhood in the city of Rio de Janeiro. Incidence was defined as the number of lab-confirmed cases per 10,000 inhabitants. (A) ZIKV incidence is greater in neighborhoods with little access to municipal water supplies in the city of Rio de Janeiro. (B) CHIKV incidence increases with the percentage of urbanized land in the neighborhood.

**Fig 4:**
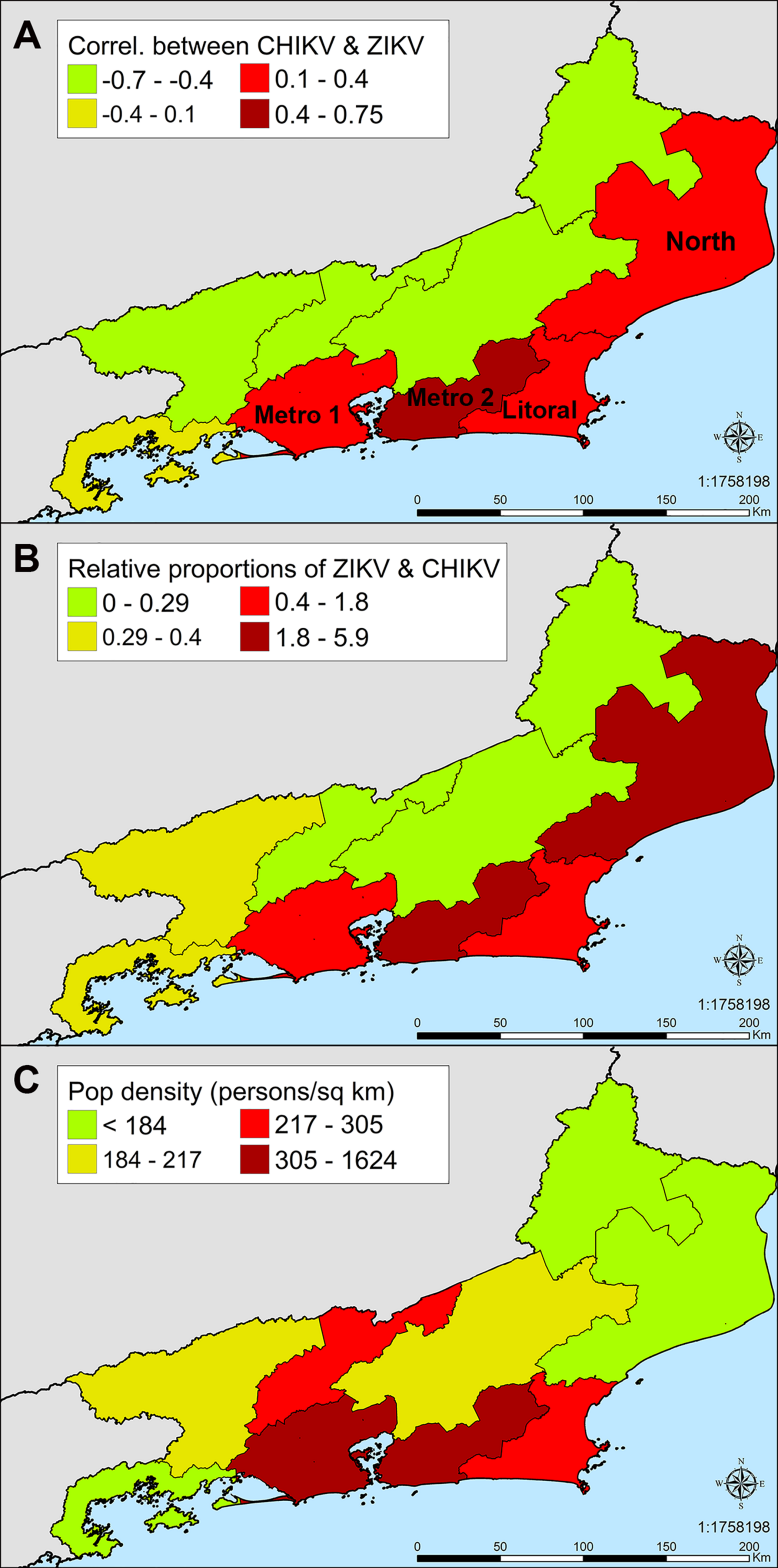
Geographic pattern of ZIKV and CHIKV incidence. (A) High correlation between the incidence of ZIKV and CHIKV in the Metro 2 health region, which comprises the eastern half of the metropolitan area of the city of Rio de Janeiro. This geographic overlap between the viruses could have led to competition in *Aedes aegypti*. The correlation is moderate but lower in the Metro 1 region. The Metro 1 and 2 regions represent 80% of the state’s population and 90% of the samples in this analysis. The correlation is also high in the North and Coastal regions, whereas in other health regions, ZIKV and CHIKV appear not to be correlated. However, the lower sample sizes outside the metropolitan area make it difficult to draw robust conclusions about the correlation in these regions. (B) Relative proportion of ZIKV and CHIKV. ZIKV dominates over CHIKV in the Rio de Janeiro metropolitan area and the Coastal and North health regions. (C) Human population density.

ZIKV incidence was inversely proportional to the percentage of households connected to municipal water infrastructure in the city of Rio de Janeiro (*r* = -0.47, *t* = -6.1, *df* = 130, *p* = 1.2 x 10^−8^, Fig 3A). There was no relationship between mosquito density and the incidence of ZIKV (*r* = 0.12, *t* = 0.899, *df* = 57, *p* = 0.37) or CHIKV (*r* = 0.035, *t* = 0.267, *df* = 57, *p* = 0.79). CHIKV incidence increased with the percent of urbanized land in each neighborhood (*r* = 0.2, *t* = 2.3, *df* = 130, *p* = 0.02, Fig 3B). Although significantly more individuals aged 40 or younger were tested than those aged 40 or older, CHIKV incidence was greater in individuals 40 years of age or older (*p*< 2.2 × 10^−16^, Table 2). ZIKV incidence was highest in individuals 20–39 years of age (4.81 cases/100,000 people). However, as ZIKV sampling was biased toward women who were pregnant, it is not possible to drawn robust conclusions about incidence in different age groups. The surveillance data also provided insights about the spatial pattern of ZIKV incidence. There was geographic overlap between health regions with high incidence of ZIKV and CHIKV, which could have led to competition of the viruses in *Aedes aegypti* (Fig 4).

**Table 2:** Cases of CHIKV and ZIKV by age among the LABFLA lab-confirmed positives.

	LABFLA lab-confirmed cases	
Age (years)	CHIKV (%)	ZIKV (%)
**0–9**	59 (2.7)	22 (1.3)
**10–19**	106 (4.9)	212 (12.3)
**20–39**	577 (26.6)	1319 (76.8)
**40–59**	903 (41.6)	137 (8)
**> 60**	525 (24.2)	27 (1.6)
**Total**	**2170**	**1717**

### Epidemic model

**Fig 5:**
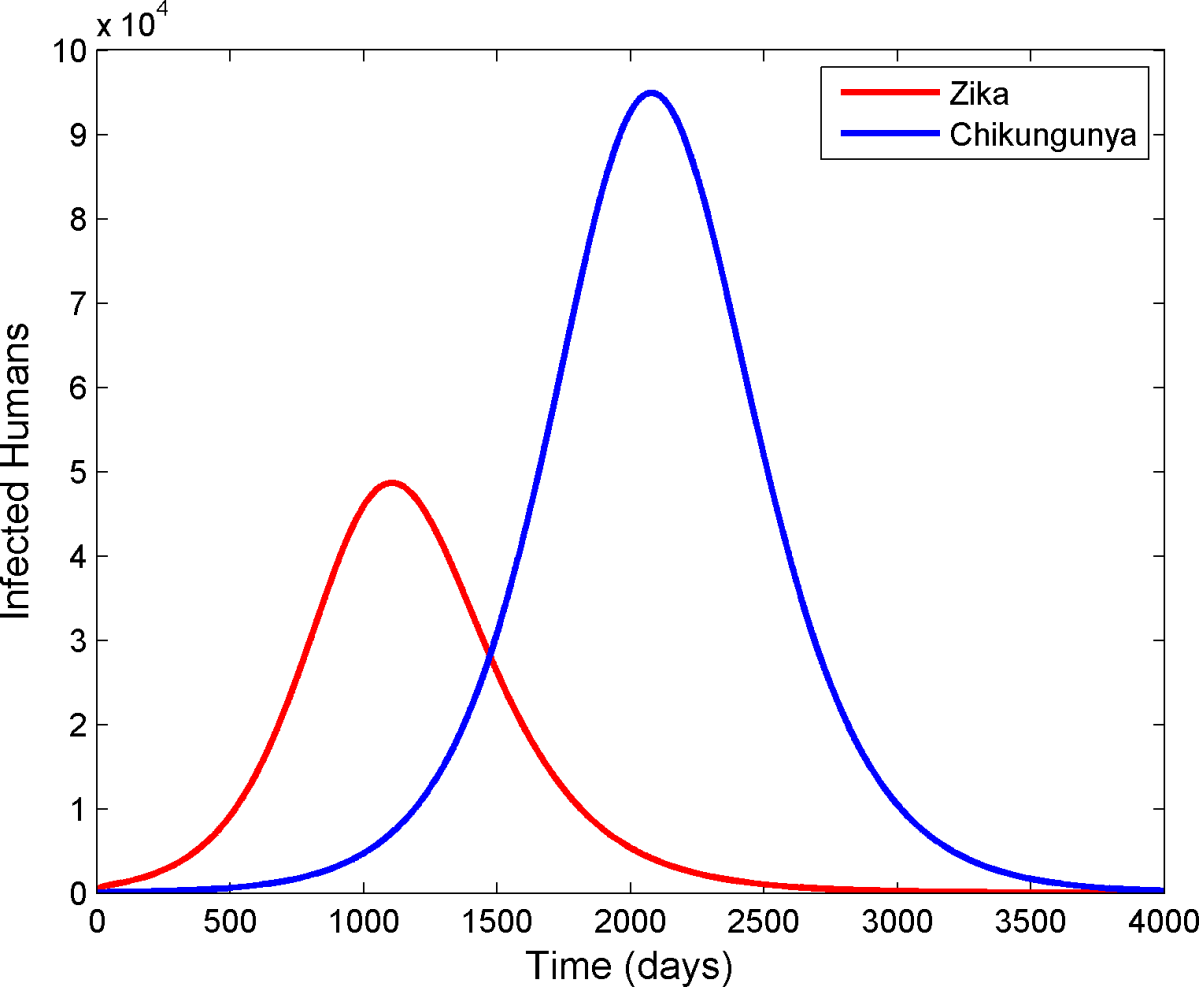
Model in which CHIKV outcompetes ZIKV is supported by the data. In the observed epidemiological data (Fig 2), the epidemic of ZIKV occurred before that of CHIKV. In our simulations, CHIKV spread more quickly than ZIKV in the mosquito, and the outbreak of ZIKV was followed by an outbreak of CHIKV.

We compared the timing of the observed epidemics to the timing of epidemics simulated using the epidemic model. Our analysis indicated that the incubation period of the viruses in the mosquito was an important parameter for determining the timing of ZIKV and CHIKV epidemics. The incubation period of CHIKV in *Aedes aegypti* is 2–4 days whereas that of ZIKV is at least 10 days (see refs. in S3 Table). When we parameterized the model with these settings, CHIKV spread more rapidly and replaced ZIKV in the simulated mosquito population. As CHIKV had a shorter incubation period than ZIKV in the mosquitoes in the simulations, it was transmitted more frequently to humans than was ZIKV, which was associated with a decline in the number of human cases of ZIKV and an increase in human cases of CHIKV. Over time, the number of cases of CHIKV also declined in the simulated human population as individuals recovered from the infection (Fig 5).

## Discussion

The CHIKV and ZIKV epidemics in Rio de Janeiro illustrate that integrating information across viruses and climatic and socioeconomic variables reveals perspectives that would not have been possible by examining one virus at a time. ZIKV did not become notifiable until February 2016. If a decision-maker only had access to data from 2016, he or she would have missed the association between rainfall and ZIKV in the last quarter of 2015. In addition, our analysis provides insights about climatic and ecological factors associated with the start and the end of the ZIKV epidemic, which could be useful for predicting and controlling future arbovirus outbreaks. We found that heavy rainfall precedes cases by three weeks and is a predictor of potential outbreaks. Rainstorms in early October 2015 likely would have increased mosquito breeding sites and the abundance of adult mosquitoes after a lag of a few weeks. The rainfall-associated increase in vector abundance may have triggered the largest ZIKV outbreak, which began in late October 2015.

In Rio de Janeiro, ZIKV and CHIKV both circulated at low prevalence in the first half of 2015, but did not trigger large epidemics. The largest wave of the ZIKV epidemic occurred in the fourth quarter of 2015 followed by the CHIKV epidemic in the first quarter of 2016, which leads to the question of why CHIKV did not spread before or at the same time as ZIKV. Modeling studies have shown that when the prevalence of an infectious disease is low, as was the case for ZIKV and CHIKV in the first half of 2015, the disease may go extinct due to demographic stochasticity. Whether the disease goes extinct or triggers an epidemic depends on factors such as the infectious period. Modeling studies indicate that if the infectious period is highly variable, there is a greater risk that the disease will go extinct [30]. The infectious period of CHIKV appears to be highly variable: while in many patients the illness resolves in ten days, approximately 50% of patients may remain symptomatic with arthralgia for up to one year [33]. The variable infectious period of CHIKV may have led to its extinction due to demographic stochasticity in 2015, when the largest wave of the ZIKV epidemic occurred. According to this scenario, CHIKV may have subsequently been reintroduced and spread in Rio de Janeiro causing an epidemic in 2016.

Modeling studies indicate that when two arboviruses are present in a human population, one virus will generally drive the other to extinction, and which one will persist is determined by factors such as the number of mosquitoes that are initially infected with each virus [34]. The shorter incubation period of CHIKV may have made it a superior competitor that was able to spread more quickly than ZIKV in the mosquito population. Other factors that could have influenced the timing of simulated epidemics include the number of mosquitoes and humans assumed to be susceptible to or infected with the two viruses at the beginning of the simulations.

Areas of high CHIKV and ZIKV incidence were not merely areas where there is always high mosquito density. Instead, it appears to be levels of urbanization and access to municipal water that contributed significantly to the CHIKV and ZIKV epidemics in the city of Rio de Janeiro. Socio-economic status (SES) may affect arbovirus risk as people with lower SES may have lifestyle factors such as living in more crowded conditions that increase arbovirus risk [35, 36]. More broadly, at the continental scale environmental factors like altitude may also influence ZIKV risk; for instance, risk appears to decrease with altitude, as high elevations are ecologically unsuitable for the mosquito [37]. CHIKV incidence was weakly correlated with the percentage of urbanized land per neighborhood but not water infrastructure. The two viruses may have been associated with distinct environmental factors because of they differ with respect to their rates of spread in the population. The basic reproduction number *R*_*0*_, which is the estimated number of new cases generated by an infected individual, is estimated to be 4 for CHIKV [38], but only 2 for ZIKV [39]. Due to its more rapid rate of spread, CHIKV may be associated with different environmental factors than ZIKV the incidence of which was lower in neighborhoods without water infrastructure.

Our finding that rainfall appears to precede ZIKV and CHIKV epidemics suggests that an early warning system based on weather that predicts these outbreaks a few weeks in advance would provide policy-makers and clinicians a warning to prepare countermeasures, which could lead to improved prognoses for ZIKV patients. For example, GBS is treated with intravenous immunoglobulin or plasmapheresis [40–42]. Physicians in tropical countries may not have immunoglobulin on hand, but a weather-based early warning system could give them a lead-time of a few weeks to gain access to the treatment. Our geographic analysis found that health regions in the Rio de Janeiro metropolitan area and the North health region of the state reported the highest ZIKV incidence and could be vulnerable to future outbreaks. The vulnerable health regions identified here could be prioritized for strengthening the capacity of the public health system to respond to ZIKV.

The analysis identified cohorts with high incidence of arbovirus infection, which could help decision-makers prioritize human populations for educational campaigns and outreach. Confirmed ZIKV cases were almost all women, which could be because women are more likely than men to seek treatment, because health authorities allocated more resources to screening samples from pregnant women due to the risk of fetal microcephaly, or because there is male-to-female sexual transmission of ZIKV that resulted in higher incidence in women [43, 44]. The rationale for our analysis of ZIKV and CHIKV incidence by age was that this was that incidence of DENV increases with the percent of women and persons older than 60 in the population, possibly because these populations have greater exposure to *Aedes aegypti* within the household [26, 45, 46]. Since CHIKV, DENV, and ZIKV are all transmitted by the same vector, we hypothesized that the incidence of ZIKV and CHIKV would be higher in women and older age groups. The incidence of CHIKV was higher in persons older than 40, which is similar to the incidence by age group in Suriname [47]. This pattern could arise because middle-aged and elderly individuals are more apt to seek healthcare, have greater exposure to *Aedes* mosquitoes because they spend more time indoors without air conditioning in developing countries, or because their general health is poorer making them more susceptible to virus infection.

Our analysis was subject to limitations that may restrict the generalizability of our findings to other geographic regions. Since ZIKV is a novel pathogen, we only had samples for a two-year period. It is possible that the climatic and environmental drivers of ZIKV in 2015 and 2016 may not be important in future years, which would limit the extent to which rainfall could be used to predict epidemics in other countries. Our results suggest that it is not merely the occurrence of rainfall that triggers ZIKV epidemics. For example, there were rainstorms in late December 2015 but ZIKV began declining during this period. Future work should investigate how the intensity and duration of rainstorms affect mosquito abundance. For instance, extremely intense rainstorms that produce a high volume of precipitation in a few hours may flush larvae leading to decreased vector abundance and arbovirus transmission [48].

One hypothesis for the decline in ZIKV disease cases in the first quarter of 2016 was that CHIKV and ZIKV competed within the vector Aedes aegypti, and CHIKV was the superior competitor and spread more quickly. An alternative hypothesis is that ZIKV infections decreased because the State Health Department implemented effective mosquito abatement programs in early 2016 that reduced the transmission of the virus, however then one should not have seen a subsequent rise in CHIKV cases which is dependent on the same vector. Yet another hypothesis is that ZIKV disease cases declined because the population became infected and acquired immunity. This hypothesis could be tested by measuring ZIKV seroprevalence. Seroprevalence surveys in the Pacific Islands found that ZIKV infected 1% of the population of New Caledonia and 12% of the population of French Polynesia [49]. As ZIKV seroprevalence studies have not yet been carried out in Brazil [50, 51], the population immunity hypothesis awaits confirmation in future studies. An additional shortcoming of the analysis is that our geographic analysis assumes that an individual was infected in the municipality where he or she resides. As *Aedes* mosquitoes are diurnal, it is possible that individuals are bitten not in the household but in the workplace, which could be located in a different municipality.

Nevertheless, our data are robust in the sense that our identification of ZIKV cases was strictly based on molecular assays, which provides a definitive diagnosis. Identification of ZIKV cases based on serology or clinical findings can result in false positive results due to serologic cross-reactivity between ZIKV and prior existing DENV antibodies in patients residing in endemic areas. Diagnosis based on clinical symptoms can also misclassify cases of ZIKV, DENV or CHIKV as the diseases have similar clinical presentations and tend to co-circulate in endemic areas. The WHO’s ZIKV Strategic Plan states that controlling arboviruses requires mapping their social and environmental drivers [52]. Our findings can contribute to such efforts in the state of Rio de Janeiro by showing that rainfall predicts arbovirus epidemics and by identifying the cohorts and geographic regions with the highest incidence. It is plausible that in the coming years other arboviruses will expand their ranges from Africa and Asia to the Americas like CHIKV and ZIKV. The development of accurate predictive models and surveillance data analysis approaches is essential if we are to prevent the next novel virus from causing a major public health disaster like ZIKV in Brazil.

## Supporting information

S1 Fig Timeline of ZIKV and CHIKV epidemics and surveillance in the state of Rio de Janeiro, 2015–2016. (DOCX)

S2 Fig Incidence of lab-confirmed ZIKV and CHIKV cases. (DOCX)

S3 Fig Timing of temperature and lab-confirmed cases of ZIKV (LABFLA dataset) January 2015-July 2016. (DOCX)

S4 Fig Timing of relative humidity and lab-confirmed cases of ZIKV (LABFLA dataset) January 2015-July 2016. (DOCX)

S1 Table Incidence of ZIKV and CHIKV infection by municipality in the state of Rio de Janeiro. (DOCX)

S2 Table Formulation of the epidemic model. (DOCX)

S3 Table ZIKV and CHIKV incubation period in *Aedes* mosquitoes. (DOCX)

S4 Table Settings used in the epidemic model simulations. (DOCX)
