# Supplementary material for: Behavioral, climatic, and environmental risk factors for Zika and Chikungunya virus infections in Rio de Janeiro, Brazil, 2015-16
Source: PLoS One. 2017 Nov 16;12(11):e0188002. doi: 10.1371/journal.pone.0188002 (PMC5690671; doi:10.1371/journal.pone.0188002)
Supplement: S1 Fig — (DOCX) [file pone.0188002.s001.docx]

**S1 Fig. Timeline of ZIKV and CHIKV epidemics and surveillance in the state of Rio de Janeiro, 2015-2016.** The largest wave of the ZIKV epidemic began in the fourth quarter of 2015 (first horizontal red bar), however, official surveillance did not begin until 2016. Thus, the unofficial surveillance by LABFLA is the only source of data on the early, exponential growth phase of the ZIKV epidemic.
