# Supplementary material for: Behavioral, climatic, and environmental risk factors for Zika and Chikungunya virus infections in Rio de Janeiro, Brazil, 2015-16
Source: PLoS One. 2017 Nov 16;12(11):e0188002. doi: 10.1371/journal.pone.0188002 (PMC5690671; doi:10.1371/journal.pone.0188002)

**S2 Fig. Incidence of lab-confirmed ZIKV and CHIKV cases.** Polygons outlined in black are the health regions of Rio de Janeiro and those outlined in gray are municipalities. In each panel incidence is divided into four quantiles from low (green) to high (red). (A) ZIKV incidence; (B) CHIKV incidence.


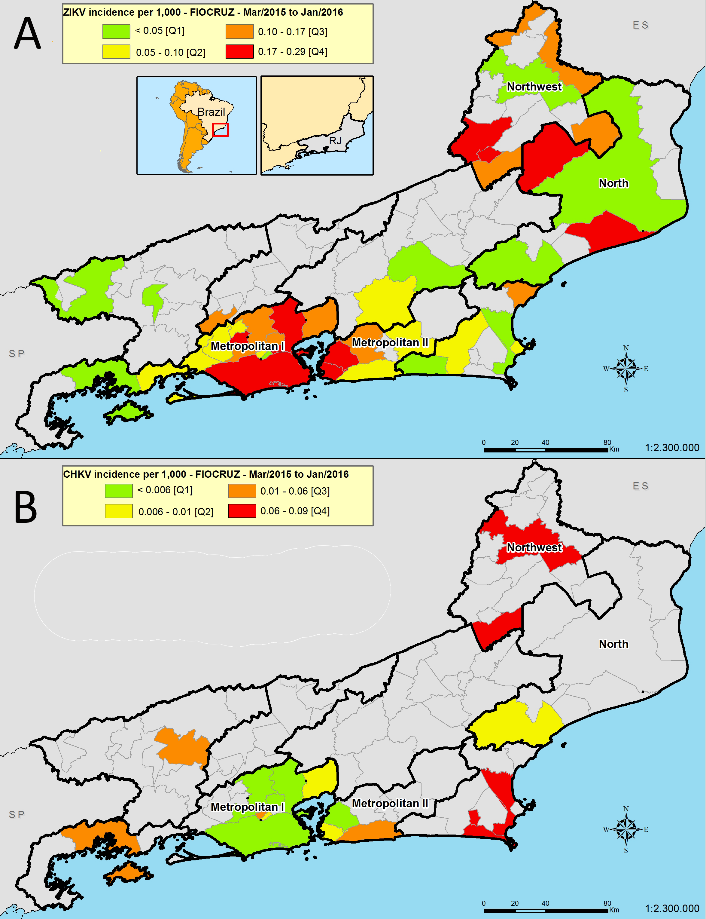

Supplement: S2 Fig — (DOCX) [file pone.0188002.s002.docx]
