# Supplementary material for: Behavioral, climatic, and environmental risk factors for Zika and Chikungunya virus infections in Rio de Janeiro, Brazil, 2015-16
Source: PLoS One. 2017 Nov 16;12(11):e0188002. doi: 10.1371/journal.pone.0188002 (PMC5690671; doi:10.1371/journal.pone.0188002)
Supplement: S4 Fig — (DOCX) [file pone.0188002.s004.docx]

**S4 Fig. Timing of relative humidity and lab-confirmed cases of ZIKV, January 2015-July 2016.** Unlike rainfall, cases of ZIKV did not appear to be correlated with relative humidity.
