# Supplementary material for: Behavioral, climatic, and environmental risk factors for Zika and Chikungunya virus infections in Rio de Janeiro, Brazil, 2015-16
Source: PLoS One. 2017 Nov 16;12(11):e0188002. doi: 10.1371/journal.pone.0188002 (PMC5690671; doi:10.1371/journal.pone.0188002)
Supplement: S1 Table — (DOCX) [file pone.0188002.s005.docx]

**S1 Table. Incidence of ZIKV and CHIKV infection by municipality in the state of Rio de Janeiro**. Incidence was calculated as the number of lab-confirmed cases per 10,000 inhabitants. Data are shown for municipalities that reported at least one case of ZIKV or CHIKV infection.

| **Municipality** | **ZIKV** | **CHIKV** |
| --- | --- | --- |
| Angra dos Reis | 0.10 | 0.45 |
| Aperibé | 0.88 | 0.00 |
| Araruama | 0.39 | 0.31 |
| Armação dos Búzios | 0.92 | 4.91 |
| Arraial do Cabo | 0.00 | 0.68 |
| Barra do Piraí | 0.10 | 0.21 |
| Barra Mansa | 0.06 | 0.00 |
| Belford Roxo | 0.76 | 0.56 |
| Bom Jesus do Itabapoana | 1.14 | 0.00 |
| Cabo Frio | 0.09 | 7.06 |
| Cachoeiras de Macacu | 0.36 | 0.00 |
| Campos dos Goytacazes | 0.80 | 0.04 |
| Cantagalo | 0.52 | 0.00 |
| Cardoso Moreira | 0.78 | 0.00 |
| Carmo | 0.52 | 0.00 |
| Duque de Caxias | 1.87 | 0.39 |
| Engenheiro Paulo de Frontin | 0.00 | 2.09 |
| Iguaba Grande | 0.00 | 1.13 |
| Itaboraí | 0.95 | 0.18 |
| Itaguaí | 0.32 | 0.24 |
| Itaocara | 0.88 | 1.32 |
| Itaperuna | 0.20 | 0.70 |
| Itatiaia | 0.00 | 0.72 |
| Japeri | 0.62 | 0.51 |
| Laje do Muriaé | 0.00 | 1.44 |
| Macaé | 0.11 | 0.15 |
| Magé | 1.20 | 0.27 |
| Mangaratiba | 0.22 | 0.22 |
| Maricá | 0.55 | 0.12 |
| Mesquita | 0.63 | 2.59 |
| Nilópolis | 0.24 | 1.52 |
| Niterói | 1.81 | 1.31 |
| Nova Friburgo | 0.21 | 0.00 |
| Nova Iguaçu | 0.95 | 0.74 |
| Paracambi | 0.77 | 0.00 |
| Paraty | 0.00 | 0.24 |
| Paty do Alferes | 0.00 | 0.39 |
| Petrópolis | 0.00 | 0.17 |
| Porciúncula | 0.53 | 2.67 |
| Queimados | 1.78 | 0.78 |
| Quissamã | 0.78 | 0.39 |
| Resende | 0.16 | 0.16 |
| Rio Bonito | 0.34 | 0.00 |
| Rio Claro | 0.58 | 0.00 |
| Rio das Ostras | 0.44 | 0.05 |
| Rio de Janeiro | 1.39 | 2.42 |
| Santo Antônio de Pádua | 1.52 | 0.00 |
| São Fidélis | 1.35 | 0.27 |
| São Gonçalo | 1.51 | 0.13 |
| São João da Barra | 14.78 | 9.67 |
| São João de Meriti | 0.02 | 0.18 |
| São Pedro da Aldeia | 0.00 | 0.20 |
| São Sebastião do Alto | 1.10 | 0.00 |
| Sapucaia | 0.59 | 0.00 |
| Saquarema | 0.57 | 0.23 |
| Seropédica | 0.49 | 0.12 |
| Teresópolis | 0.06 | 0.28 |
| Valença | 0.00 | 0.27 |
| Volta Redonda | 0.04 | 0.08 |
