# Supplementary material for: Behavioral, climatic, and environmental risk factors for Zika and Chikungunya virus infections in Rio de Janeiro, Brazil, 2015-16
Source: PLoS One. 2017 Nov 16;12(11):e0188002. doi: 10.1371/journal.pone.0188002 (PMC5690671; doi:10.1371/journal.pone.0188002)
Supplement: S2 Table — (DOCX) [file pone.0188002.s006.docx]

**S2 Table. Formulation of the epidemic model.**

| **Indices/Sets** | **Description** | |
| --- | --- | --- |
| $i\in\left\{ 1,2 \right\}$ | Index for the two arboviruses: ZIKV = 1, CHIKV=2 | |
| $S_{H}^{i}$ | Humans susceptible to arbovirus $i$ | |
| $I_{H}^{i}$ | Humans infected with arbovirus $i$ | |
| $R_{H}^{i}$ | Humans recovered from arbovirus $i$ | |
| $S_{V}^{i}$ | *Aedes aegypti* mosquito population susceptible to arbovirus $i$ | |
| $E_{V}^{i}$ | Mosquito population exposed to arbovirus $i$ but not yet infectious | |
| $I_{V}^{i}$ | Mosquito population infected with arbovirus $i$ and capable of transmitting to humans | |
|  |  | |
| **Parameters** | **Description** | |
| $\beta_{HV}^{i}$ | Probability of transmission of arbovirus $i$ from mosquitos to humans | |
| $\nu_{H}$ | Human natality rate | |
| $\mu_{H}$ | Human mortality rate | |
| $\gamma^{i}$ | Recovery rate of humans infected with arbovirus $i$ | |
| $\beta_{VH}^{i}$ | Probability of transmission of arbovirus $i$ from humans to mosquitos | |
| $\nu_{V}$ | Mosquito recruitment rate | |
| $\mu_{V}$ | Mosquito mortality rate | |
| $r$ | Mosquito bite rate | |
| $\alpha^{i}$ | Extrinsic incubation period of arbovirus $i$ in the mosquito | |
| $N_{H}$ | Human population size | |
|  | | |
|  | | |
| **Formulation** | | |
| $\frac{{dS}_{H}^{i}}{dt}=\nu_{H}-{r\frac{\beta_{VH}^{i}}{N_{H}}S}_{H}^{i}I_{V}^{i}-\mu_{H}S_{H}^{i}$ | | (1) |
| $\frac{dI_{H}^{i}}{dt}=r\frac{\beta_{VH}^{i}}{N_{H}}S_{H}^{i}I_{V}^{i}-(\gamma^{i}+\mu_{H})I_{H}^{i}$ | | (2) |
| $\frac{{dR}_{H}}{dt}=\gamma^{i}I_{H}^{i}-\mu_{H}R_{H}$ | | (3) |
| $\frac{{dS}_{V}^{i}}{dt}=\nu_{V}-r\frac{\beta_{HV}^{i}}{N_{H}}S_{V}^{i}I_{H}^{i}-\mu_{V}S_{V}^{i}$ | | (4) |
| $\frac{{dE}_{V}^{i}}{dt}=r\frac{\beta_{HV}^{i}}{N_{H}}S_{V}^{i}I_{H}^{i}-{(\alpha}^{i}+\mu_{V})E_{V}^{i}$ | | (5) |
| $\frac{dI_{V}^{i}}{dt}=\alpha^{i}E_{V}^{i}-\mu_{V}I_{V}^{i}$ | | (6) |
