# Supplementary material for: Behavioral, climatic, and environmental risk factors for Zika and Chikungunya virus infections in Rio de Janeiro, Brazil, 2015-16
Source: PLoS One. 2017 Nov 16;12(11):e0188002. doi: 10.1371/journal.pone.0188002 (PMC5690671; doi:10.1371/journal.pone.0188002)
Supplement: S3 Table — (DOCX) [file pone.0188002.s007.docx]

**S3 Table. ZIKV and CHIKV incubation period in *Aedes* mosquitoes.**

| **Virus** | **Incubation period (d)** | **Ref.** |
| --- | --- | --- |
| CHIKV | 2 | 1 |
|  | 3 | 2 |
|  | 4 | 3 |
| ZIKV | 10 | 2 |
|  | 14 | 4 |
|  | 15 | 5 |

**References**

1. Dong S, Kantor AM, Lin J, Passarelli AL, Clem RJ, Franz AWE. Infection pattern and transmission potential of Chikungunya virus in two New World laboratory-adapted *Aedes aegypti* strains. Scientific Reports. 2016;6:24729. doi: 10.1038/srep24729.

2. Manore CA, Ostfeld RS, Agusto FB, Gaff H, LaDeau SL. Defining the risk of Zika and Chikungunya virus transmission in human population centers of the eastern United States. PLoS Neglected Tropical Diseases. 2017;11(1):e0005255. doi: 10.1371/journal.pntd.0005255.

3. Manore CA, Hickmann KS, Xu S, Wearing HJ, Hyman JM. Comparing dengue and Chikungunya emergence and endemic transmission in *A. aegypti* and *A. albopictus*. Journal of Theoretical Biology. 2014;356:174-91. doi: 10.1016/j.jtbi.2014.04.033.

4. Chouin-Carneiro T, Vega-Rua A, Vazeille M, Yebakima A, Girod R, Goindin D, et al. Differential susceptibilities of *Aedes aegypti* and *Aedes albopictus* from the Americas to Zika Virus. PLoS Neglected Tropical Diseases. 2016;10(3):e0004543. doi: 10.1371/journal.pntd.0004543. PubMed PMID: MEDLINE:26938868.

5. Musso D, Gubler DJ. Zika Virus. Clinical Microbiology Reviews. 2016;29(3):487-524. doi: 10.1128/CMR.00072-15.
