# Supplementary material for: Behavioral, climatic, and environmental risk factors for Zika and Chikungunya virus infections in Rio de Janeiro, Brazil, 2015-16
Source: PLoS One. 2017 Nov 16;12(11):e0188002. doi: 10.1371/journal.pone.0188002 (PMC5690671; doi:10.1371/journal.pone.0188002)
Supplement: S4 Table — (DOCX) [file pone.0188002.s008.docx]

**S4 Table. Settings used in the epidemic model simulations.**

| **Parameter** | **Definition** | **Value** | **Ref.** |
| --- | --- | --- | --- |
| $\beta_{HV}^{ZIKV}$ | Probability of transmission of ZIKV from mosquito to human | 0.7 | 1 |
| $\nu_{H}$ | Human natality rate | 50 | Assumed |
| $\mu_{H}$ | Human mortality rate | 0.000055 | 2 |
| $\gamma^{ZIKV}$ | Recovery rate | 0.01 | 3 |
| $\beta_{VH}^{ZIKV}$ | Probability of transmission of ZIKV from human to mosquito | 0.7 | 1 |
| $\mu_{V}$ | Mosquito mortality rate | 0.05 | 4 |
| $\nu_{V}$ | Mosquito recruitment rate | 50000 | Assumed |
| $r$ | Mosquito bite rate | 0.05 | Assumed |
| $\gamma^{CHIKV}$ | Recovery rate | 0.02 | 4 |
| $\beta_{HV}^{CHIKV}$ | Probability of transmission of CHIKV from an infected human to a mosquito | 0.8 | 4 |
| $\beta_{VH}^{CHIKV}$ | Probability of transmission of CHIKV from an infected mosquito to a human | 0.8 | 4 |
| $\alpha^{ZIKV}$ | ZIKV extrinsic incubation period, i.e. latent period before the mosquito becomes infectious | 0.12 | 1 |
| $\alpha^{CHIKV}$ | CHIKV extrinsic incubation period | 0.6 | S3 Table |

**References**

1. Ferguson NM, Cucunuba ZM, Dorigatti I, Nedjati-Gilani GL, Donnelly CA, Basanez M-G, et al. Countering Zika in Latin America. Science. 2016;353(6297):353-4. doi: 10.1126/science.aag0219.

2. Keeling M, Rohani P. Modeling Infectious Diseases in Humans and Animals. Princeton, New Jersey: Princeton University Press; 2008.

3. Bonyah E, Okosun KO. Mathematical modeling of Zika virus. Asian Pacific Journal of Tropical Disease. 2016;6(9):673-9. doi: 10.1016/S2222-1808(16)61108-8.

4. Yakob L, Clements ACA. A mathematical model of Chikungunya dynamics and control: the major epidemic on Réunion Island. PLoS One. 2013;8(3):e57448. doi: 10.1371/journal.pone.0057448.
